# Characteristics and Quality of Mobile Apps Containing Prenatal Genetic Testing Information: Systematic App Store Search and Assessment

**DOI:** 10.2196/30404

**Published:** 2021-10-14

**Authors:** Ko-Lin Wu, Rebeca Alegria, Jazzlyn Gonzalez, Harrison Hu, Haocen Wang, Robin Page, Patricia Robbins-Furman, Ping Ma, Tung-Sung Tseng, Lei-Shih Chen

**Affiliations:** 1 Graduate School of Biomedical Sciences University of North Texas Health Science Center Fort Worth, TX United States; 2 Department of Health and Kinesiology Texas A&M University College Station, TX United States; 3 College of Veterinary Medicine & Biomedical Sciences Texas A&M University College Station, TX United States; 4 College of Science Texas A&M University College Station, TX United States; 5 College of Nursing Texas A&M University College Station, TX United States; 6 Department of Molecular and Human Genetics Baylor College of Medicine Houston, TX United States; 7 Department of Health Promotion & Community Health Sciences School of Public Health Texas A&M University College Station, TX United States; 8 Behavioral and Community Health Sciences School of Public Health Louisiana State University Health Sciences Center New Orleans, LA United States

**Keywords:** mobile applications, prenatal genetic testing, pregnancy, review, evaluation

## Abstract

**Background:**

Prenatal genetic testing is an essential part of routine prenatal care. Yet, obstetricians often lack the time to provide comprehensive prenatal genetic testing education to their patients. Pregnant women lack prenatal genetic testing knowledge, which may hinder informed decision-making during their pregnancies. Due to the rapid growth of technology, mobile apps are a potentially valuable educational tool through which pregnant women can learn about prenatal genetic testing and improve the quality of their communication with obstetricians. The characteristics, quality, and number of available apps containing prenatal genetic testing information are, however, unknown.

**Objective:**

This study aims to conduct a firstreview to identify, evaluate, and summarize currently available mobile apps that contain prenatal genetic testing information using a systematic approach.

**Methods:**

We searched both the Apple App Store and Google Play for mobile apps containing prenatal genetic testing information. The quality of apps was assessed based on the criteria adopted from two commonly used and validated mobile app scoring systems, including the Mobile Application Rating Scale (MARS) and the APPLICATIONS evaluation criteria.

**Results:**

A total of 64 mobile apps were identified. Of these, only 2 apps were developed for a specific prenatal genetic test. All others were either pregnancy-related (61/64, 95%) or genetics-related (1/64, 2%) apps that provided prenatal genetic testing information. The majority of the apps (49/64, 77%) were developed by commercial companies. The mean quality assessment score of the included apps was 13.5 (SD 2.9), which was equal to the average of possible theoretical score. Overall, the main weaknesses of mobile apps in this review included the limited number of prenatal genetic tests mentioned; incomprehensiveness of testing information; unreliable and missing information sources; absence of developmental testing with users (not evidence based); high level of readability; and the lack of visual information, customization, and a text search field.

**Conclusions:**

Our findings suggest that the quality of mobile apps with prenatal genetic testing information must be improved and that pregnant women should be cautious when using these apps for prenatal genetic testing information. Obstetricians should carefully examine mobile apps before referring any of them to their patients for use as an educational tool. Both improving the quality of existing mobile apps, and developing new, evidence-based, high-quality mobile apps targeting all prenatal genetic tests should be the focus of mobile app developers going forward.

## Introduction

Prenatal genetic testing, a set of genetic tests used to detect potential fetal disease risk, is an essential part of routine prenatal care [1,2]. Information provided by these tests allows pregnant women to make informed decisions about their pregnancy, including preparation for affected births, early management of infants with genetic disorders, and termination of affected pregnancies [1,3-5]. Although prenatal genetic testing is important for pregnant women, obstetricians often have insufficient time to provide comprehensive education about prenatal genetic testing in prenatal care [6,7]. Studies have found that pregnant women lack prenatal genetic testing knowledge, which may hinder informed decision-making during their pregnancies [4,8,9].

Mobile apps have been used to assist patients in accessing health information, facilitate engagement with their physicians, and strengthen patient-provider communication and relationships [10-12]. Lay people also perceive health information provided by apps to be accurate and trustworthy [13]. Among apps with medical information, pregnancy-related apps are the most common [14,15]. Nevertheless, the characteristics, quality, and number of available apps containing prenatal genetic testing information are unknown.

To fill this knowledge gap, we used a systematic approach to identify existing apps containing prenatal genetic testing information and summarize their characteristics. We then adapted existing app evaluation tools [16-18] to evaluate the quality of these apps. We believe our findings will have the potential to help obstetricians become familiar with prenatal genetic testing apps and make recommendations to their patients. Our results can assist researchers and app developers to recognize the strengths and limitations of existing apps that contain information pertaining to prenatal genetic testing, as well as to design apps that better meet the needs of pregnant women.

## Methods

### Overview

Because this study did not involve risk to human subjects, it was exempted from institutional review board oversight at Texas A&M University. A prenatal genetic counselor at a large medical center and a certified health education specialist who is an expert in prenatal genetic education guided, oversaw, and reviewed the research process and findings.

### App Search

**Figure 1:**
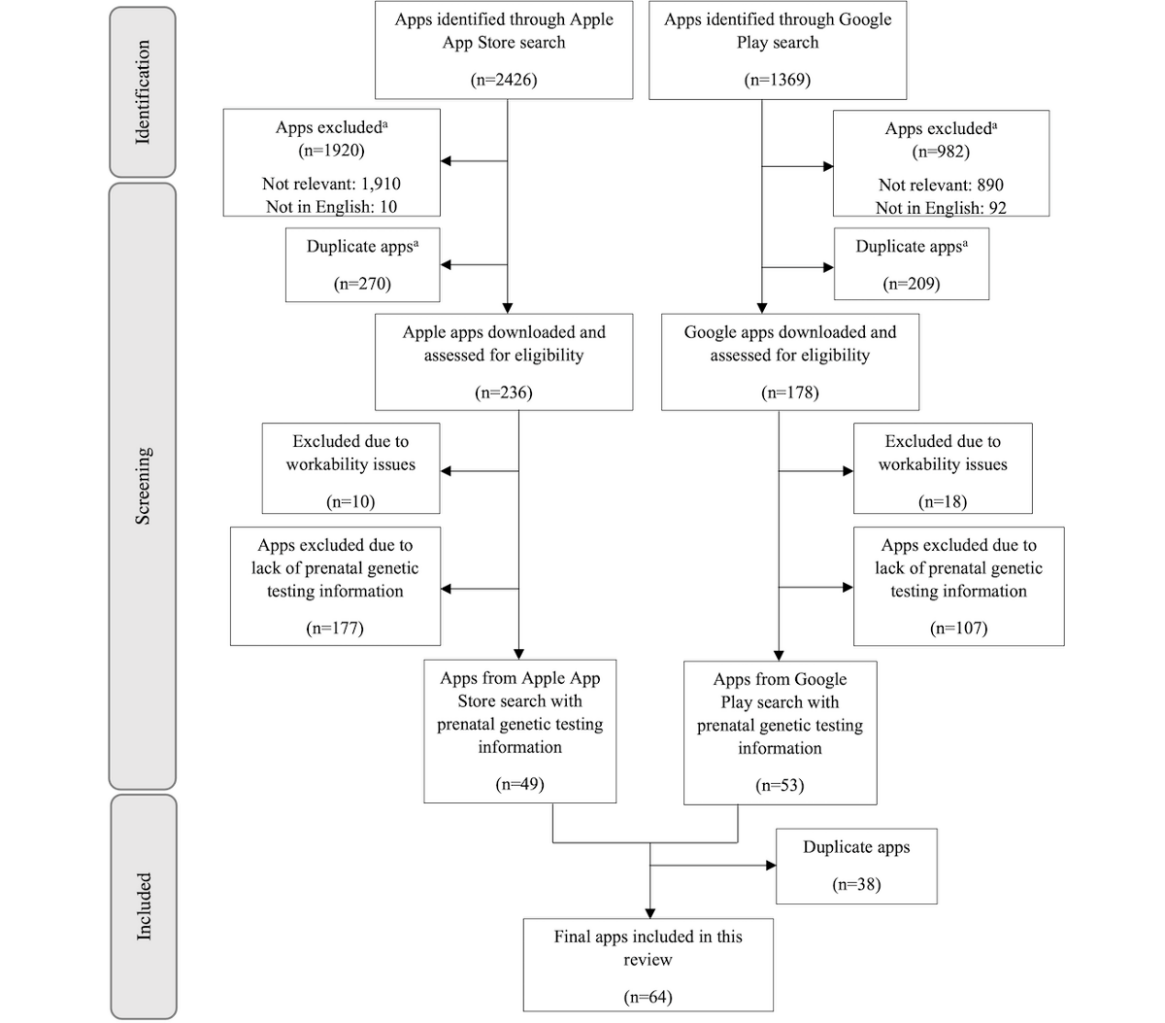
The selection process of mobile apps containing prenatal genetic testing information. ^a^The apps were excluded by reading the app titles and descriptions.

Figure 1 summarizes the search, screening, and selection procedures for the eligible prenatal genetic testing apps. Specifically, this process included four steps. First, 3 members of the research team (KW, RA, and JG) conducted a search on both the Apple App Store (iOS) and Google Play (Android) for apps with prenatal genetic testing information, between January 28, 2020, and March 23, 2020. The following search terms were used: “pregnancy,” “prenatal,” “pregnant,” “genetic testing,” “genetic screening,” “pregnancy screening,” “prenatal screening,” “prenatal screening test,” “prenatal diagnostic test,” “carrier testing,” “expanded carrier screening,” “amniocentesis,” “chorionic villus sampling,” “non-invasive prenatal testing,” “non-invasive prenatal screening,” “cell free DNA screening,” “cell free DNA testing,” “MaterniT21,” “Harmony,” “Panorama,” “QNatal,” “percutaneous umbilical blood sampling,” “cordocentesis,” “nuchal translucency scan,” “nuchal translucency ultrasound,” “quad screen,” “quadruple screen,” “quadruple test,” “first-trimester screening,” “second-trimester screening,” “modified sequential screening,” “sequential screening,” “alpha-fetoprotein screening,” “maternal serum screening,” “prenatal genetic screening test,” “prenatal genetic screening,” and “prenatal genetic testing”.

A total of 3795 apps were identified. In the second step, we used very few exclusion criteria for the initial screening in order to maximize the number of potential apps selected. A total of 2902 apps were excluded using the following exclusion criteria: (1) apps that were not relevant to prenatal genetic testing, pregnancy, and/or genetics, and (2) apps that were not in English. In the third step, we excluded apps that were duplicated within the Apple App Store (iOS) and in Google Play (Android). In the fourth step, a total of 414 apps were downloaded and thoroughly assessed. Among these, 28 apps were excluded because of workability issues, such as apps that could not be successfully downloaded or opened, and/or those that needed a specific user account from a hospital or clinic for access. Moreover, 284 apps were excluded due to the lack of prenatal genetic testing information, and 38 apps were excluded because of their duplication in both the Apple App Store and Google Play. Thus, our final sample consisted of 64 apps.

### Data Extraction

The entire research team assessed, extracted, and summarized detailed information from the included 64 apps (Multimedia Appendix 1). Extracted information included app name, operating system (ie, iOS or Android), app description, types of prenatal genetic testing mentioned in those apps, prenatal genetic test procedures mentioned therein, information on the timing of prenatal genetic tests, reliability or accuracy of prenatal genetic tests included, interpretation of the results of those prenatal genetic tests, specific disorders mentioned, prenatal genetic testing information citations, customer ratings, and cost. Moreover, according to the Mobile Application Rating Scale (MARS) app classification [18], we also extracted the app developer information (ie, details of the commercial company, hospital, or nongovernmental organization). Finally, based on the content of the apps, we utilized the Flesch-Kincaid grade level assessment to determine the readability level of the apps selected for further analysis.

### App Quality Assessment

To evaluate the quality of the selected apps, we developed the App Quality Assessment Scoring System (AQASS) by adapting two common app evaluation tools—MARS [18] and the “APPLICATIONS” evaluation criteria [16,17]. The AQASS was used to assess 16 items in each app, including the number of prenatal genetic tests mentioned, comprehensiveness, information quality, quality of information sources, evidence base, readability, visual prenatal genetic testing information, customization, interactivity, text search field, ease of navigation, app layout, visual appeal, interdevice compatibility, connectivity, and price. Table 1 lists the detailed criteria for each item in AQASS. Possible AQASS scores ranged from 0 to 27, with a higher score representing higher app quality. Following the reliability checking procedure outlined in previous research [19,20], 2 raters on the team (RA and HH) used the AQASS criteria to independently score each app, after which interrater reliability was assessed using the Gwet AC1 calculation [21]. Findings suggest a strong interrater reliability between the two raters with a coefficient of 0.86 [22].

**Table 1:** App Quality Assessment Scoring System (AQASS) ratings of mobile apps (N=64) containing prenatal genetic testing information.

Criterion and description		AQASS score	Frequency distribution (%)
**Number of prenatal genetic tests mentioned**			
	Using the general term “(prenatal) genetic testing” only	0	3
	1-5 prenatal genetic tests	1	67
	6-10 prenatal genetic tests	2	30
	All 10 prenatal genetic tests	3	0
**Comprehensiveness of information**			
	Only mentioned the name of the prenatal genetic test	0	2
	Having information in 1 or 2 of the following 5 categories: test procedures, timing of the tests, reliability or accuracy of the tests, interpretation of test results, and specific diseases or conditions to be tested for	1	25
	Having information in 3 or 4 of the following 5 categories: test procedures, timing of the tests, reliability or accuracy of the tests, interpretation of test results, and specific diseases or conditions to be tested for	2	38
	Having information in all of the following categories: test procedures, timing of the tests, reliability or accuracy of the tests, interpretation of test results, and specific diseases or conditions to be tested for	3	36
**Quality of information**			
	Information too general to assess quality	0	3
	Most information is incorrect	1	2
	Some incorrect and/or out-of-date information	2	25
	Correct and up-to-date information	3	70
**Reliable information sources**			
	No reference identified	0	75
	Questionable/unreliable source(s)	1	3
	Source from hospitals, specific obstetricians-gynecologists, or peer reviewed articles	2	13
	Source from authoritative organizations, such as the American College of Obstetricians and Gynecologists	3	9
**Evidence base**			
	App had not been trialed or tested	0	98
	App had been tested (eg, acceptability, usability, and satisfaction ratings) and had positive outcomes in studies that were not randomized control trials (RCTs), and there was no contradictory evidence.	1	2
	App had been trialed and outcome tested in 1-2 RCTs indicating positive results	2	0
	App had been trialed and outcome tested in >3 high quality RCTs indicating positive results	3	0
**Readability^a^**			
	Not applicable^b^	0	3
	>6th grade	1	95
	≤6th grade	2	2
**Visual information^c^**			
	Absent	0	92
	Present	1	8
**Customization^d^**			
	Absent	0	100
	Present	1	0
**Interactivity^e^**			
	Absent	0	31
	Present	1	69
**Text search function**			
	Absent	0	61
	Present	1	39
**Navigation ease**			
	Hard to navigate	0	14
	Easy to navigate	1	86
**Layout of the app**			
	Inappropriate, unclear, and/or some options difficult to select, locate, find, or read	0	3
	Appropriate, clear, and able to select, locate, find, or read items	1	97
**Visual appeal**			
	Unprofessional or unappealing	0	2
	Professional and appealing	1	98
**Interdevice compatibility**			
	iPhone or Android phone	0	41
	iPhone and Android phone	1	59
**Connectivity**			
	Internet not required	0	58
	Internet required	1	42
**Price**			
	Paid	0	0
	Free	1	100

^a^Readability was assessed by Flesch-Kincaid grade level assessment. According to Weiss [23], mobile health apps are recommended to have a reading level of sixth grade or below for general public use.

^b^App did not have enough text and/or complete sentences to allow for the calculation of Flesch-Kincaid grade level for readability.

^c^Visual information was defined as the presence in the app of photos, images, tables, figures, and videos to demonstrate pregame genetic tests

^d^Customization referred to a function that allows users to input prenatal genetic test results, testing dates, and other information in settings or preferences of the mobile app.

^e^Interactivity allowed users to provide feedback to the developers, offer reminders on the timing of prenatal genetic testing, have a chatroom function, and allow sharing information onto the social media.

## Results

Among 64 evaluated apps, only 2 (3.1%) apps were specifically developed for prenatal genetic testing. NIPT Insights, developed by Five Minutes Ltd., introduced noninvasive prenatal testing (NIPT) and compared it with first-trimester screening, triple/quad screening, anatomy ultrasound, chorionic villus sampling (CVS), and amniocentesis. Similarly, cfDNA Predictive Value Calculator (Perinatal Quality Foundation) offered links to a number of scientific articles regarding NIPT, and it provided users with the ability to calculate both positive and negative predictive values of NIPT. All other apps that provided prenatal genetic testing information were either pregnancy-related (61/64, 95%) or genetics (1/64, 2%) apps.

Table 2 summarizes the key characteristics of those 64 apps. Specifically, all 64 apps could be downloaded at no cost. Of these, 5 (8%) offered an optional upgrade, which were downloaded and assessed. However, upgrading did not provide any further prenatal genetic testing information. The majority of the apps (49/64, 77%) were created by commercial companies, and over half of the apps (38/64, 59%) were compatible with both the Android and iOS operating systems. Only 52 (81%) apps had a customer rating. With a theoretical range of 1 to 5 stars, the mean rating was 4.5 (SD 0.6; range 2.0-5.0).

**Table 2:** Descriptive statistics of the mobile apps (N=64) containing prenatal genetic testing information.

Characteristic and category				Value, n (%)				
**Type of developer**								
	App created by commercial companies			49 (77)				
	App created by hospitals			11 (17)				
	App created by non-governmental organizations			3 (5)				
	Apps created by the government (ie, California Department of Public Health)			1 (2)				
**Operating system**								
	Android only			15 (23)				
	iOS only			11 (17)				
	Android and iOS			38 (59)				
**Type of prenatal genetic tests mentioned**								
	Amniocentesis			49 (77)				
	CVS^a^			49 (77)				
	First trimester screening (NT^b^ screening)			46 (72)				
	Triple/quad screening			44 (69)				
	Cell-free DNA testing or NIPT^c^			35 (55)				
	Anatomy ultrasound			26 (41)				
	Cordocentesis			10 (16)				
	Expanded carrier screening			8 (13)				
	First trimester screening (blood test)			8 (13)				
	Genetic screening (prenatal) in general			7 (11)				
	Carrier screening			6 (9)				
**Prenatal genetic test procedures mentioned**								
	Amniocentesis			37 (58)				
	CVS			36 (56)				
	First trimester screening (NT screening)			34 (53)				
	Cell-free DNA testing or NIPT			25 (39)				
	Triple/quad screening			17 (27)				
	Cordocentesis			9 (14)				
	Anatomy ultrasound			5 (8)				
	Carrier screening			4 (6)				
	First trimester screening (blood test)			3 (5)				
	Expanded carrier screening			1 (2)				
	Not reported			17 (27)				
**Timing of prenatal genetic tests mentioned**								
	CVS			45 (70)				
	Amniocentesis			44 (69)				
	First trimester screening (NT screening)			40 (63)				
	Triple/quad screening			38 (59)				
	Cell-free DNA testing or NIPT			31 (48)				
	Anatomy ultrasound			23 (36)				
	Cordocentesis			9 (14)				
	First trimester screening (blood test)			8 (13)				
	Carrier screening			3 (5)				
	Expanded carrier screening			1 (2)				
	Genetic screening (prenatal) in general			1 (2)				
	Not reported			5 (8)				
**Reliability or accuracy of prenatal genetic tests mentioned**								
	Cell-free DNA testing or NIPT			23 (36)				
	Amniocentesis			21 (33)				
	First trimester screening (NT screening)			13 (20)				
	CVS			12 (19)				
	Triple/quad screening			12 (19)				
	Cordocentesis			2 (3)				
	Carrier screening			1 (2)				
	Expanded carrier screening			1 (2)				
	First trimester screening (blood test)			1 (2)				
	Anatomy ultrasound			1 (2)				
	Not reported			33 (52)				
**Interpretation of potential results of prenatal genetic tests**								
	Triple/quad screening			19 (30)				
	Cell-free DNA testing or NIPT			13 (20)				
	Amniocentesis			12 (19)				
	First trimester screening (NT screening)			9 (14)				
	Expanded carrier screening			8 (13)				
	CVS			8 (13)				
	Carrier screening			3 (5)				
	Genetic screening (prenatal) in general			1 (2)				
	Not reported			36 (56)				
**Specific disorder(s) mentioned**								
	Trisomy 21 (Down syndrome)			53 (83)				
	Trisomy 18 (Edward syndrome)			33 (52)				
	Cystic fibrosis			29 (45)				
	Trisomy 13 (Patau syndrome)			18 (28)				
	Sickle cell anemia			14 (22)				
	Tay-Sachs disease			11 (17)				
	Spinal muscular atrophy			8 (13)				
	**Neural tube defect (general)**			3 (5)				
		Spina bifida	15 (23)					
		Anencephaly	6 (9)					
	Thalassemia			3 (5)				
	Fragile X syndrome			2 (3)				
	Huntington disease			2 (3)				
	Muscular dystrophy			2 (3)				
	Klinefelter syndrome			1 (2)				
	Prader-Willi syndrome			1 (2)				
	Trisomy 16			1 (2)				
	Turner syndrome			1 (2)				
	Not reported			9 (14)				
**Citation for prenatal genetic testing information**								
	No			48 (75)				
	Yes (reliable source)			14 (22)				
	Yes (non-reliable source)			2 (3)				
Cost^d^: $0				64 (100)				
Customer rating^e^, mean (SD); range				4.5 (0.6); 2.0-5.0				

^a^CVS: chorionic villus sampling.

^b^NT: nuchal translucency.

^c^NIPT: noninvasive prenatal testing.

^d^Five apps had an optional upgrade fee but upgrading did not affect the amount of prenatal genetic testing information available to the user.

^e^Only 52 of the 64 apps (81.3%) had customer ratings.

A total of 10 different types of prenatal genetic tests were mentioned across all apps, including carrier screening, expanded carrier screening, first-trimester screening (blood test), first-trimester screening (nuchal translucency screening), cell-free DNA testing or NIPT, CVS, amniocentesis, triple/quad screening, anatomy ultrasound, and cordocentesis/percutaneous umbilical blood sampling. Amniocentesis and CVS were most common types of prenatal genetic tests referred to in apps (49/64, 77% for both), whereas carrier screening (6/64, 9%) was the least mentioned.

All apps specifically referred to 17 genetic disorders tested for by prenatal genetic testing; these included testing for the following disorders: trisomy 21 (53/64, 83%), trisomy 18 (33/64, 52%), cystic fibrosis (29/64, 45%), trisomy 13 (18/64, 28%), spina bifida (15/64, 23%), sickle cell anemia (14/64, 22%), Tay-Sachs disease (11/64, 17%), spinal muscular atrophy (8/64, 13%), anencephaly (6/64, 9%), neural tube defect as a general term (3/64, 5%), thalassemia (3/64, 5%), Fragile X syndrome (2/64, 3%), Huntington disease (2/64, 3%), muscular dystrophy (2/64, 3%), Klinefelter syndrome (1/64, 2%), Prader-Willi syndrome (1/64, 2%), trisomy 16 (1/64, 2%), and Turner syndrome (1/64, 2%). Nevertheless, 14.1% (9/64) of the apps did not mention any genetic disorders that could be identified by prenatal genetic testing. Information that was missing in the reviewed apps included context about the interpretation of test results (36/64, 56%), the reliability or the accuracy of prenatal genetic tests (33/64, 52%), testing procedures (17/64, 27%), and testing time (5/64, 8%). In terms of references and citations found in these apps, only 14 of 64 apps (22%) included reliable sources (eg, the Mayo Clinic, the American Pregnancy Association, and the American College of Obstetricians and Gynecologists [ACOG]) to support their prenatal genetic testing information.

Table 1 summarizes the AQASS ratings for the quality of the 64 apps we evaluated. With a possible score range of 0 to 27, the mean score for all apps was 13.5 (SD 2.89; range 5-18). In particular, a majority of the apps were found to be visually appealing (63/64, 98%), have appropriate and clear layouts (62/64, 97%), easy to navigate (55/64, 86%), contain interactivity functions (44/64, 69%), and require no internet to access once downloaded (37/64, 58%). Nevertheless, none of the apps included all types of prenatal genetic tests. Most apps (43/64, 67%) reported only 1 to 5 types of prenatal genetic tests. Only 23 (36%) of all the evaluated apps addressed procedures, timing, reliability or accuracy, and interpretation of the results of the prenatal genetic tests. Although 45 (70%) of the apps presented correct and up-to-date information, merely 8 apps (13%) provided reliable information sources from hospitals, specific obstetricians-gynecologists, and/or peer reviewed articles, or referenced authoritative organizations such as the ACOG (6/64, 9%). Furthermore, a large percentage of the apps had a reading level higher than the sixth grade (61/64, 95%), did not include any visual information for prenatal genetic testing (eg, photos, images, tables, figures, and videos) (59/64, 92%), and lacked a text search field (39/64, 61%). None of the apps had a customization function to allow for the tailoring of the prenatal genetic information to the patients’ circumstances (eg, inputting gestational weeks as well as prenatal genetic testing dates and results in settings or preferences). Nearly all apps (63/64, 98%) had not been tested by research studies to determine their quality and efficacy.

## Discussion

### Principal Findings

It is important for pregnant women to understand prenatal genetic testing in order to make informed decisions about whether or not to utilize these tests during their pregnancy [4]. Apps are a potential educational tool to help pregnant women understand such complex information [24]. To our knowledge, our study is the first systematic review of apps containing prenatal genetic testing information. We found 64 apps currently available that provide information about prenatal genetic testing. Nevertheless, none of those apps presented comprehensive information about all prenatal genetic tests.

Our results showed that commercial companies had developed the majority of the 64 apps with prenatal genetic testing information we found. We also noticed that approximately one-third of all evaluated apps contained incorrect and/or out-of-date information. It is recommended that developers in commercial companies collaborate with a team of health care professionals, including obstetricians, geneticists, and genetic counselors to design apps with accurate and up-to-date information. Moreover, due to the rapid advancement of genomic technologies, information and guidelines regarding prenatal genetic testing may evolve periodically [25]. Thus, developers should review their apps constantly and update information as needed.

The average AQASS score of the 64 apps containing prenatal genetic testing information for our final sample was 13.5, which was equal to the mean score of the possible theoretical AQASS score (range 0-27). This low score was in line with a previous review that suggested that the quality of genetics and genomics apps that need improvement [19]. The low scores of the apps we evaluated was mainly due to the limited number of prenatal genetic tests mentioned, incomprehensiveness of testing information, unreliable and missing information sources; absence of developmental and clinical testing with users; high readability levels; and the lack of visual information presented, customization options, and a text search field.

Unfortunately, the regulation of health apps has not been well established in the United States. Although the Food and Drug Administration (FDA) has published a policy regulating medical health apps, only mobile apps classified as medical devices are currently under federal oversight and require the FDA approval before they are released to the public [26,27]. Because the FDA does not regulate health apps focused on providing health information [26,27], the often-low quality we found in these apps that include information of prenatal genetic testing can cause pregnant women to be exposed to incomplete, unreliable information regarding prenatal genetic testing. As such, comprehensive regulatory standards and guidelines for these apps are urgently required.

### Clinical and Research Implications

Our study has important clinical and research implications. First, due to the lack of high-quality and comprehensive apps that provide prenatal genetic testing information, we caution obstetricians to be prepared to address the questions raised by patients who may have previously obtained incorrect and incomplete information from those apps. Second, obstetricians should also be aware of the limitations of the apps with prenatal genetic testing information when communicating with their patients. Moreover, establishing policies to regulate apps that include prenatal genetic testing information is warranted. Finally, from both research and clinical standpoints, the creation of a high-quality app specifically designed for prenatal genetic testing that has been evaluated using a randomized control trial to test its efficacy in improving pregnant women’s prenatal genetic testing decision-making is recommended.

### Strengths and Limitations

There are several limitations to this study. As we only reviewed apps with information on prenatal genetic testing in English, there may be apps developed in languages other than English that were not included in this review. Similarly, our app search was conducted in the United States. Future researchers may consider changing platform and search settings to include apps available in countries outside the United States to allow for a broader search. Furthermore, as a team, we carefully conducted the app search together and asked 3 additional researchers to independently check our search results. Some apps, however, may have been overlooked. As the app search was concluded on March 23, 2020, new apps with prenatal genetic testing information may have become available after that date.

Despite these limitations, this study has a number of strengths. It is a unique study and the first of its kind attempting to identify apps containing prenatal genetic testing information. After screening a total of 3795 apps on the Apple App Store and Google Play platforms, we identified 64 apps. In addition, we also carefully examined each app to present a thorough summary of the information they contained. Beyond reporting the characteristics of the identified apps, we developed the AQASS to capture a detailed overview of the quality of the apps we reviewed, as most apps were not created with the primary purpose of conveying prenatal genetic testing to pregnant women. Our AQASS was based on commonly used and validated app scoring systems (ie, MARS and APPLICATIONS) [16-18]. We adopted both these systems because several items of MARS (eg, “Quality of information: Is app content correct, well written, and relevant to the goal/topic of the app?”) and APPLICATIONS (eg, “advertisements” and “subjective presentation”) were not applicable to the evaluation of the apps that provide prenatal genetic testing information in our study. Conversely, some items from either MARS (eg, “Evidence base: Has the app been trialed/tested?”) or APPLICATIONS (eg, “connectivity” and “navigation ease”) were found to be suitable for the evaluation of apps that include information on prenatal genetic testing. Beyond the use of AQASS for our study, it can also be adopted to assess other apps that were not originally designed to primarily address the specific topic under evaluation. It should be noted that the reliability and validity of AQASS also need to be further examined in the future.

### Conclusions

This study is the first to extensively search apps containing information on prenatal genetic testing in both the Apple App Store and Google Play, summarize their characteristics, and assess their quality. We identified 64 available apps containing information about prenatal genetic testing and found that the quality of those apps needs to be improved. Additionally, none of the apps we evaluated were specifically designed to introduce all prenatal genetic tests. As such, pregnant women should be cautious when using these apps for prenatal genetic testing information. Obstetricians should carefully examine apps before any recommendation is made for their use as an educational tool. Improving the quality of existing apps and developing new, evidence-based, high-quality apps with a targeted focus on prenatal genetic testing is strongly recommended in the future.

## Appendix

Multimedia Appendix 1 Characteristics of the reviewed mobile apps.

## Abbreviations

ACOG: American College of Obstetricians and Gynecologists
AQASS: App Quality Assessment Scoring System
CVS: chorionic villus sampling
FDA: Food and Drug Administration
MARS: Mobile Application Rating Scale
