# Supplementary material for: Characteristics and Quality of Mobile Apps Containing Prenatal Genetic Testing Information: Systematic App Store Search and Assessment
Source: JMIR Mhealth Uhealth. 2021 Oct 14;9(10):e30404. doi: 10.2196/30404 (PMC8554671; doi:10.2196/30404)
Supplement: Multimedia Appendix 1 [file mhealth_v9i10e30404_app1.docx]

**Appendix 1. Characteristics of the 64 Apps with Prenatal Genetic Testing Information**

| **App name (version/last updated month and year)** | | **Developer (type of developer)** | **Operating system** | **Brief description** | **Readability level: Flesch-Kincaid (FK) grade level** | **Types of prenatal genetic tests mentioned** | **Prenatal genetic testing procedures mentioned** | **Prenatal genetic tests timing mentioned** | **Prenatal genetic test reliability/ accuracy mentioned** | **Prenatal genetic testing results explained** | **Specific disorders mentioned** | **Citations accompanying prenatal genetic testing information** | **Customer rating** | **Cost** | **AQASS Score^m^** |
| --- | --- | --- | --- | --- | --- | --- | --- | --- | --- | --- | --- | --- | --- | --- | --- |
| 2life baby^a^  (1.0.20/March 2018) | | 2 For Life Media Inc. (commercial company) | iOS (iPhone) | A basic pregnancy app that estimates the weight of the fetus week-by-week. | - FK grade level: 10.4 | - First-trimester screening (NT screening) - Triple/Quad screening - Anatomy ultrasound - Cell-free DNA testing/NIPT - Amniocentesis - CVS | - First-trimester screening (NT screening) - Triple/Quad screening - Amniocentesis | - First-trimester screening (NT screening) - Triple/Quad screening - Anatomy ultrasound - Cell-free DNA testing/NIPT - Amniocentesis - CVS | - Amniocentesis - Triple/Quad screening | Not reported | - Cystic fibrosis - Neural tube defect (Spina bifida) - Trisomy 18 (Edward’s Syndrome) - Trisomy 21 (Down Syndrome) | Yes (reliable source) | 5.0/5.0 | Free | 15 |
| Ava Fertility Tracker  (1.10.0/January 2020) | Ava AG (commercial company) | | iOS (iPhone) and Android | This app offers week-by-week information on pregnancy body, baby status, a “what to do” section, and a checkups section that includes prenatal genetic testing information. | - FK grade level: 10.8 | - First-trimester screening (NT screening) - Triple/Quad screening - Cell-free DNA testing/NIPT - Amniocentesis - CVS | - First-trimester screening (NT screening) - Cell-free DNA testing/NIPT - Amniocentesis - CVS | - First-trimester screening (NT screening) - Triple/Quad screening - Cell-free DNA testing/NIPT - Amniocentesis - CVS | - Cell-free DNA testing/NIPT - CVS | Not reported | - Cystic fibrosis - Muscular dystrophy - Neural tube defect (Spina bifida) - Sickle cell anemia - Tay-Sachs disease - Trisomy 13 (Patau Syndrome) - Trisomy 18 (Edward’s Syndrome) - Trisomy 21 (Down Syndrome) | No | 4.3/5.0 | Free | 13 |
| Baby Yourself (2.31/August 2019) | UTIC Insurance Company (commercial company) | | iOS (iPhone) and Android | A pregnancy week-by-week app that includes checklists, tips, trackers, and a journal. Tips section includes many detailed articles. | - FK grade level: 12.8 | - Triple/Quad screening - Anatomy ultrasound - Cell-free DNA testing/NIPT - Amniocentesis - CVS | - Triple/Quad screening - Amniocentesis - CVS | - Amniocentesis - CVS | - Triple/Quad screening - Amniocentesis - CVS | - Triple/Quad screening - CVS | - Cystic fibrosis - Neural tube defect (Anencephaly) - Neural tube defect (Spina bifida) - Trisomy 18 (Edward’s Syndrome) - Trisomy 21 (Down Syndrome) | Yes (reliable source) | N/A^b^ | Free | 17 |
| Babychakra Pregnancy Baby App^c^  (5.4.2/March 2019) | Infomoko Technology Pvt. Ltd. (commercial company) | | iOS (iPhone) and Android | A weekly pregnancy app based in India that allows users to engage with its in-app community. App includes a shopping section as well as many articles regarding baby growth, pregnancy symptoms, beauty, and style. | - FK grade level: 13.7 | - Triple/Quad screening - Amniocentesis - CVS | - CVS | - Triple/Quad screening - Amniocentesis - CVS | - CVS | Not reported | - Neural tube defect (Anencephaly) - Neural tube defect (Spina bifida) - Trisomy 21 (Down Syndrome) | No | 3.0/5.0 | Free | 16 |
| Babynote- Pregnancy Timeline (2.3/March 2019) | HYPE4MOBILE MALEWICZ (commercial company) | | iOS (iPhone) | A weekly pregnancy app that tell users the shape of the baby, health tips, and tips on medical advice during pregnancy. | - FK grade level: 5 | - Anatomy ultrasound | Not reported | - Anatomy ultrasound | Not reported | Not reported | - Trisomy 18 (Edward’s Syndrome) - Trisomy 21 (Down Syndrome) | No | 4.4/5.0 | Free | 9 |
| BHealth Baby-The Baby App from Baptist Health System (1.2/March 2016) | Sunny Media Group (commercial company) | | iOS (iPhone) and Android | An app with tips, tracking methods and week-by-week information for pregnant women. Includes very limited prenatal genetic testing information. | - FK grade level: 11.3 | - Triple/Quad Screening | Not reported | - Triple/Quad Screening | Not reported | - Triple/Quad screening | Not reported | No | 4.8/5 | Free | 10 |
| cfDNA Predictive Value Calculator^a^  (1.0.0/September 2016) | Perinatal Quality Foundation (commercial company) | | Android | An app that provides information about certain genetic testing and has a calculator tool which calculates positive and negative predictive values. | - FK grade level: 13.9 | - Cell-free DNA testing/NIPT | Not reported | Not reported | - Cell-free DNA testing/NIPT | - Cell-free DNA testing/NIPT | - Trisomy 18 (Edward’s Syndrome) - Trisomy 21 (Down Syndrome) | Yes (reliable source) | N/A^b^ | Free | 14 |
| Circle by Covenant Health^d^ (4.0.1654/March 2020) | Covenant Health System (hospital) | | iOS (iPhone) and Android | A health app that has many articles regarding preventative care, nutrition, week-by-week pregnancy information, and A-Z symptoms of pregnancy. It includes dedicated articles on prenatal genetic testing. | - FK grade level: 11 | - Expanded carrier screening - First-trimester screening (NT screening) - Triple/Quad screening - Anatomy ultrasound - Cell-free DNA testing/NIPT - Amniocentesis - CVS - Cordocentesis/ Percutaneous umbilical blood sampling (PUBS) | - First-trimester screening (NT screening) - Cell-free DNA testing/NIPT - Amniocentesis - CVS - Cordocentesis/ Percutaneous umbilical blood sampling (PUBS) | - First-trimester screening (NT screening) - Triple/Quad screening - Anatomy ultrasound - Cell-free DNA testing/NIPT - Amniocentesis - CVS - Cordocentesis/ Percutaneous umbilical blood sampling (PUBS) | - First-trimester screening (NT screening) - Cell-free DNA testing/NIPT - Amniocentesis | - Expanded carrier screening - Triple/Quad screening - Cell-free DNA testing/NIPT - Amniocentesis | - Cystic fibrosis - Spinal muscular atrophy - Trisomy 18 (Edward’s Syndrome) - Trisomy 21 (Down Syndrome) | No | 4.9/5.0 | Free | 15 |
| Circle by Hoag^d^ (4.0.1654/March 2020) | Hoag Memorial Hospital Presbyterian (hospital) | | iOS (iPhone) and Android | A health app that has many articles regarding preventative care, nutrition, week-by-week pregnancy information, and A-Z symptoms of pregnancy. It also includes dedicated articles on prenatal genetic testing. | - FK grade level: 10.8 | - Expanded carrier screening - First-trimester screening (NT screening) - Triple/Quad screening - Anatomy ultrasound - Cell-free DNA testing/NIPT - Amniocentesis - CVS - Cordocentesis/ Percutaneous umbilical blood sampling (PUBS) | - First-trimester screening (NT screening) - Cell-free DNA testing/NIPT - Amniocentesis - CVS - Cordocentesis/ Percutaneous umbilical blood sampling (PUBS) | - First-trimester screening (NT screening) - Triple/Quad screening - Anatomy ultrasound - Cell-free DNA testing/NIPT - Amniocentesis - CVS - Cordocentesis/ Percutaneous umbilical blood sampling (PUBS) | - First-trimester screening (NT screening) - Cell-free DNA testing/NIPT - Amniocentesis | - Expanded carrier screening - Triple/Quad screening - Cell-free DNA testing/NIPT - Amniocentesis | - Cystic fibrosis - Spinal muscular atrophy - Trisomy 18 (Edward’s Syndrome) - Trisomy 21 (Down Syndrome) | No | 4.9/5/0 | Free | 15 |
| Circle by Kadlec^d^ (4.0.1654/March 2020) | Kadlec (hospital) | | iOS (iPhone) and Android | A health app that has many articles regarding preventative care, nutrition, week-by-week pregnancy information, and A-Z symptoms of pregnancy. It also includes dedicated articles on prenatal genetic testing. | - FK grade level: 10.8 | - Expanded carrier screening - First-trimester screening (NT screening) - Triple/Quad screening - Anatomy ultrasound - Cell-free DNA testing/NIPT - Amniocentesis - CVS - Cordocentesis/ Percutaneous umbilical blood sampling (PUBS) | - First-trimester screening (NT screening) - Cell-free DNA testing/NIPT - Amniocentesis - CVS - Cordocentesis/ Percutaneous umbilical blood sampling (PUBS) | - First-trimester screening (NT screening) - Triple/Quad screening - Anatomy ultrasound - Cell-free DNA testing/NIPT - Amniocentesis - CVS - Cordocentesis/ Percutaneous umbilical blood sampling (PUBS) | - First-trimester screening (NT screening) - Cell-free DNA testing/NIPT - Amniocentesis | - Expanded carrier screening - Triple/Quad screening - Cell-free DNA testing/NIPT - Amniocentesis | - Cystic fibrosis - Spinal muscular atrophy - Trisomy 18 (Edward’s Syndrome) - Trisomy 21 (Down Syndrome) | No | 4.8/5.0 | Free | 15 |
| Circle by OSF^d^ (4.0.1654/March 2020) | OSF Healthcare System (hospital) | | iOS (iPhone) and Android | A health app that has many articles regarding preventative care, nutrition, week-by-week pregnancy information, and A-Z symptoms of pregnancy. It also includes dedicated articles on prenatal genetic testing. | - FK grade level: 10.5 | - Expanded carrier screening - First-trimester screening (NT screening) - Triple/Quad screening - Anatomy ultrasound - Cell-free DNA testing/NIPT - Amniocentesis - CVS - Cordocentesis/ Percutaneous umbilical blood sampling (PUBS) | - First-trimester screening (NT screening) - Cell-free DNA testing/NIPT - Amniocentesis - CVS - Cordocentesis/ Percutaneous umbilical blood sampling (PUBS) | - First-trimester screening (NT screening) - Triple/Quad screening - Anatomy ultrasound - Cell-free DNA testing/NIPT - Amniocentesis - CVS - Cordocentesis/ Percutaneous umbilical blood sampling (PUBS) | - First-trimester screening (NT screening) - Cell-free DNA testing/NIPT - Amniocentesis | - Expanded carrier screening - Triple/Quad screening - Cell-free DNA testing/NIPT - Amniocentesis | - Cystic fibrosis - Spinal muscular atrophy - Trisomy 18 (Edward’s Syndrome) - Trisomy 21 (Down Syndrome) | No | 4.7/5.0 | Free | 15 |
| Circle by Providence^d^  (3.1/October 2019) | Providence Health & Services - Washington (hospital) | | iOS (iPhone) and Android | A health app that has many articles regarding preventative care, nutrition, week-by-week pregnancy information, and A-Z symptoms of pregnancy. It also includes dedicated articles on prenatal genetic testing. | - FK grade level: 9.9 | - Expanded carrier screening - First-trimester screening (NT screening) - Triple/Quad screening - Anatomy ultrasound - Cell-free DNA testing/NIPT - Amniocentesis - CVS - Cordocentesis/ Percutaneous umbilical blood sampling (PUBS) | - First-trimester screening (NT screening) - Cell-free DNA testing/NIPT - Amniocentesis - CVS - Cordocentesis/ Percutaneous umbilical blood sampling (PUBS) | - First-trimester screening (NT screening) - Triple/Quad screening - Anatomy ultrasound - Cell-free DNA testing/NIPT - Amniocentesis - CVS - Cordocentesis/ Percutaneous umbilical blood sampling (PUBS) | - First-trimester screening (NT screening) - Cell-free DNA testing/NIPT - Amniocentesis | - Expanded carrier screening - Triple/Quad screening - Cell-free DNA testing/NIPT - Amniocentesis | - Cystic fibrosis - Spinal muscular atrophy - Trisomy 18 (Edward’s Syndrome) - Trisomy 21 (Down Syndrome) | No | 4.8/5.0 | Free | 15 |
| Circle by St. Joseph Health^d^ (4.0.1654/March 2020) | St. Joseph Health (hospital) | | iOS (iPhone) and Android | A health app that has many articles regarding preventative care, nutrition, week-by-week pregnancy information, and A-Z symptoms of pregnancy. It also includes dedicated articles on prenatal genetic testing. | - FK grade level: 9.5 | - Expanded carrier screening - First-trimester screening (NT screening) - Triple/Quad screening - Anatomy ultrasound - Cell-free DNA testing/NIPT - Amniocentesis - CVS - Cordocentesis/ Percutaneous umbilical blood sampling (PUBS) | - First-trimester screening (NT screening) - Cell-free DNA testing/NIPT - Amniocentesis - CVS - Cordocentesis/ Percutaneous umbilical blood sampling (PUBS) | - First-trimester screening (NT screening) - Triple/Quad screening - Anatomy ultrasound - Cell-free DNA testing/NIPT - Amniocentesis - CVS - Cordocentesis/ Percutaneous umbilical blood sampling (PUBS) | - First-trimester screening (NT screening) - Cell-free DNA testing/NIPT - Amniocentesis | - Expanded carrier screening - Triple/Quad screening - Cell-free DNA testing/NIPT - Amniocentesis | - Cystic fibrosis - Spinal muscular atrophy - Trisomy 18 (Edward’s Syndrome) - Trisomy 21 (Down Syndrome) | No | 4.8/5.0 | Free | 15 |
| Circle by Swedish^d^ (3.1/October 2019) | Swedish Health Services (hospital) | | iOS (iPhone) and Android | A health app that has many articles regarding preventative care, nutrition, week-by-week pregnancy information, and A-Z symptoms of pregnancy. It also includes dedicated articles on prenatal genetic testing. | - FK grade level: 8.8 | - Expanded carrier screening - First-trimester screening (NT screening) - Triple/Quad screening - Anatomy ultrasound - Cell-free DNA testing/NIPT - Amniocentesis - CVS - Cordocentesis/ Percutaneous umbilical blood sampling (PUBS) | - First-trimester screening (NT screening) - Cell-free DNA testing/NIPT - Amniocentesis - CVS - Cordocentesis/ Percutaneous umbilical blood sampling (PUBS) | - First-trimester screening (NT screening) - Triple/Quad screening - Anatomy ultrasound - Cell-free DNA testing/NIPT - Amniocentesis - CVS - Cordocentesis/ Percutaneous umbilical blood sampling (PUBS) | - First-trimester screening (NT screening) - Cell-free DNA testing/NIPT - Amniocentesis | - Expanded carrier screening - Triple/Quad screening - Cell-free DNA testing/NIPT - Amniocentesis | - Cystic fibrosis - Spinal muscular atrophy - Trisomy 18 (Edward’s Syndrome) - Trisomy 21 (Down Syndrome) | No | 4.8/5.0 | Free | 15 |
| DueDatePlusbyBSC-Pr  (4.0.1655/April 2020) | Wildflower Health (commercial company) | | iOS (iPhone) | A pregnancy app for expecting mothers that provides them with resources and important information for a healthy pregnancy, including basic prenatal genetic testing information. | - FK grade level: 5.8 | - Cell-free DNA testing/NIPT - Triple/Quad Screening - CVS - First-trimester screening (blood test) | - Cell-free DNA testing/NIPT - Triple/Quad Screening - CVS - First-trimester screening (blood test) | - Cell-free DNA testing/NIPT - Triple/Quad Screening - CVS - First-trimester screening (blood test) | - Not reported | - Triple/Quad Screening | - Trisomy 21 (Down Syndrome) - Neural tube defect (Spina bifida) | Yes (reliable resources) | 5.0/5.0 | Free | 16 |
| Femometer Fertility Tracker (3.15.1/March 2020) | Hangzhou Bangtang Network Technology Co., Ltd. (commercial company) | | iOS (iPhone) and Android | An app that includes menstrual period tracking to help users get pregnant faster or avoid getting pregnant. Additional features contain: a diary to track weight gains, articles relating to exercise, diet, symptoms, and limited information on prenatal genetic testing. | - FK grade level: 9.2 | - (Prenatal) genetic screening in general - First-trimester screening (NT screening) - Triple/Quad screening - Amniocentesis - CVS | - First-trimester screening (NT screening) | - First-trimester screening (NT screening) - Triple/Quad screening | Not reported | - (Prenatal) genetic screening in general | - Trisomy 13 (Patau Syndrome) - Trisomy 18 (Edward’s Syndrome) - Trisomy 21 (Down Syndrome) | No | 4.9/5.0 | Free  ($10 monthly subscription upgrade)^e^ | 13 |
| Fertility, Ovulation App & Pregnancy Tracker^f^  (3.4/March 2020) | ElaWoman-Period Tracker & Ovulation Calendar App  (commercial company) | | iOS (iPhone) and Android | A week-by-week app that provides numerous articles with relatable information for every week during pregnancy and minimal prenatal genetic testing information. | - FK grade level: 9.4 | - (Prenatal) genetic screening in general - First-trimester screening (NT screening) - Triple/Quad screening - Cell-free DNA testing/NIPT - Amniocentesis | - First-trimester screening (NT screening) - Triple/Quad screening - Cell-free DNA testing/NIPT - Amniocentesis | - First-trimester screening (NT screening) - Triple/Quad screening - Cell-free DNA testing/NIPT - Amniocentesis | Not reported | Not reported | - Trisomy 13 (Patau Syndrome) - Trisomy 18 (Edward’s Syndrome) - Trisomy 21 (Down Syndrome) | No | 4.2/5.0 | Free | 13 |
| FirstCry India (7.0.0/April 2020) | Digital Age Retail Private Limited (commercial company) | | iOS (iPhone) and Android | This app has 2 parts: parenting section and shopping section. The parenting section has an expecting section which is based on pregnancy weeks, which includes “pregnancy inspection schedule.” | - FK grade level: 9.9 | - Triple/quad screening - Anatomy ultrasound - Cell-free DNA testing/NIPT - Amniocentesis - CVS | - Triple/Quad screening - Anatomy ultrasound - Amniocentesis - CVS | - Triple/Quad screening - Anatomy ultrasound - Cell-free DNA testing/NIPT - Amniocentesis - CVS | - Amniocentesis - CVS | - Triple/Quad screening - Amniocentesis - CVS | - Cystic fibrosis - Neural tube defect (Anencephaly) - Neural tube defect (Spina bifida) - Sickle cell anemia - Tay-Sachs disease - Trisomy 13 (Patau Syndrome) - Trisomy 18 (Edward’s Syndrome) - Trisomy 21 (Down Syndrome) | No | 4.6/5.0 | Free | 16 |
| FLO (4.63/April 2020) | Flo Health, Inc. (commercial company) | | iOS (iPhone) and Android | A menstrual period and pregnancy app that contains a health insights section with articles and courses on various topics including prenatal genetic testing. | - FK grade level: 12.3 | - Carrier screening - First-trimester screening (blood test) - First-trimester screening (NT screening) - Triple/Quad screening - Anatomy ultrasound - Cell-free DNA testing/NIPT - Amniocentesis - CVS | - Amniocentesis | - First-trimester screening (blood test) - First-trimester screening (NT screening) - Triple/Quad screening - Anatomy ultrasound - Cell-free DNA testing/NIPT - Amniocentesis - CVS | Not reported | Not reported | - Cystic fibrosis - Sickle cell anemia - Tay-Sachs disease - Trisomy 13 (Patau Syndrome) - Trisomy 18 (Edward’s Syndrome) - Trisomy 21 (Down Syndrome) | Yes (reliable source) | 4.8/5.0 | Free ($4.17 monthly subscription upgrade)^e^ | 17 |
| Get Pregnant: Fertility Days (N/A)^g^ | Gor Aghavelyan (commercial company) | | iOS (iPhone) | An app designed to help women get pregnant by tracking ovulation and also provides tips for already pregnant women. It provides limited information on prenatal genetic testing. | - FK grade level: 12.1 | - (Prenatal) genetic screening in general | Not reported | Not reported | Not reported | Not reported | - Cystic Fibrosis - Sickle cell disease | No | 3.7/5.0 | Free | 5 |
| GLOW: Pregnancy & Baby Tracker (3.7.13/April 2020) | Glow Inc. (commercial company) | | iOS (iPhone) and Android | A week-by-week app that lets users track pregnancy symptoms and has community groups for advice. | - FK grade level: 8.8 | - First-trimester screening (NT screening) - Anatomy ultrasound - Triple/Quad screening - Amniocentesis - CVS | - First-trimester screening (NT screening) - Amniocentesis - CVS | - First-trimester screening (NT screening) - Triple/Quad screening - Amniocentesis - CVS | - First-trimester screening (NT screening) - Amniocentesis | - First-trimester screening (NT screening) - Anatomy ultrasound | - Neural tube defect (Anencephaly) - Neural tube defect (Spina bifida) - Trisomy 21 (Down Syndrome) - Trisomy 18 (Edward’s Syndrome) | No | 4.5/5.0 | Free ($4 monthly subscription upgrade)^e^ | 16 |
| I’m Pregnant-Pregnancy Week By Week  (4.0/March 2020) | BabyJoyApp (commercial company) | | Android | A week-by-week app with multiple tracking devices and minimal prenatal genetic testing information. | - FK grade level: 9.1 | - First-trimester screening (NT screening) - Triple/Quad screening - Amniocentesis - CVS | Not reported | - First-trimester screening (NT screening) - Triple/Quad screening - Amniocentesis - CVS | Not reported | Not reported | - Trisomy 21 (Down Syndrome) | No | 4.7/5.0 | Free | 12 |
| Indian Pregnancy Advice, Baby Care, Parenting Tips (2.69/March 2020) | Parentune - Parenting, Child care Growth Tracker (commercial company) | | Android | A week-by-week app with multiple articles for specific topics and extensive prenatal genetic testing information. | - FK grade level: 9.4 | - First-trimester screening (NT screening) - Triple/Quad screening - Cell-free DNA testing/NIPT - Amniocentesis - CVS - Cordocentesis/ Percutaneous umbilical blood sampling (PUBS) | - First-trimester screening (NT screening) - Cell-free DNA testing/NIPT - Amniocentesis | - Cell-free DNA testing/NIPT - Amniocentesis - CVS | - Triple/Quad screening - Cell-free DNA testing/NIPT - Amniocentesis - Cordocentesis/ Percutaneous umbilical blood sampling (PUBS) | Not reported | - Cystic fibrosis - Fragile X Syndrome - Huntington's disease - Neural tube defect (general) - Sickle cell anemia - Trisomy 21 (Down Syndrome) | Yes (reliable source) | 4.6/5.0 | Free | 16 |
| Mama Natural (1.8/April 2020) | Tinh Van Trinh (commercial company) | | iOS (iPhone) | The app features articles on pregnancy, baby, parenting, and general lifestyle. It has articles on screening tests done in each trimester of pregnancy. | - FK grade level: 11.7 | - First-trimester screening (blood test) - First-trimester screening (NT screening) - Triple/Quad screening - Anatomy ultrasound - Cell-free DNA testing/NIPT - Amniocentesis - CVS | - First-trimester screening (blood test) - First-trimester screening (NT screening) - Triple/Quad screening - Anatomy ultrasound - Cell-free DNA testing/NIPT - Amniocentesis - CVS | - First-trimester screening (blood test) - First-trimester screening (NT screening) - Triple/Quad screening - Anatomy ultrasound - Cell-free DNA testing/NIPT - Amniocentesis - CVS | - Triple/Quad screening - Cell-free DNA testing/NIPT - Amniocentesis | - CVS | - Neural tube defect (Anencephaly) - Neural tube defect (Spina bifida) - Trisomy 13 (Patau Syndrome) - Trisomy 18 (Edward’s Syndrome) - Trisomy 21 (Down Syndrome) | No | 4.8/5.0 | Free | 15 |
| Mendelian Genetics  (1.0/March 2015) | Wiki Kids Limited (commercial company) | | Android | An app that describes the Mendel approach to genetics and includes minimal prenatal genetic testing information. | - FK grade level: 14.7 | - Amniocentesis - CVS | - Amniocentesis - CVS | - Amniocentesis - CVS | Not reported | Not reported | - Tay-Sachs Disease | No | 4.2/5.0 | Free | 10 |
| Monogram Maternity (1.1.4/October 2019) | Ascension Health (non-governmental organization) | | iOS (iPhone) and Android | An app that has information that covers pregnancy, labor and birth, postpartum, breastfeeding, and newborn care. It offers educational content that includes some prenatal genetic testing information. | - FK grade level: 11.3 | - Triple/Quad screening - Cell-free DNA testing/NIPT - Amniocentesis - CVS | Not reported | - Triple/Quad screening - Cell-free DNA testing/NIPT - CVS | Not reported | Not reported | Not reported | No | N/A^b^ | Free | 13 |
| mum & baby  (4.0.2/February 2020) | Imagineear (commercial company) | | iOS (iPhone) | An app designed to provide information about pregnancy and early childcare. | - FK grade level: 8.9 | - First-trimester screening (blood test) - Anatomy ultrasound | Not reported | - First-trimester screening (blood test) - Anatomy ultrasound | Not reported | Not reported | Not reported | No | N/A^b^ | Free | 12 |
| my baby. By Dignity Health  (4.0.1649/February 2020) | Dignity Health (non-governmental organization) | | iOS (iPhone) and Android | A weekly pregnancy app that offers community resources and pregnancy education programs. | - FK grade level: 8 | - First-trimester screening (NT screening) - Triple/Quad screening - Cell-free DNA testing/NIPT - CVS | - First-trimester screening (NT screening) - CVS | - First-trimester screening (NT screening) - Triple/Quad screening - CVS | Not reported | Not reported | - Cystic fibrosis - Neural tube defect (Spina bifida) - Trisomy 18 (Edward’s Syndrome) - Trisomy 21 (Down Syndrome) | No | 3.8/5.0 | Free | 14 |
| MyPregnancy@ (2.0.0/March 2019) | Horizon Strategic Partners Ltd. (hospital) | | iOS (iPhone) and Android | A hospital affiliated app that offers information on early and later pregnancy, problems during pregnancy, and advice after birth. | - FK grade level: 9 | - First-trimester screening (NT screening) - Triple/Quad screening - Cell-free DNA testing/NIPT - Amniocentesis - CVS | - First-trimester screening (NT screening) - Triple/Quad screening - Cell-free DNA testing/NIPT - Amniocentesis - CVS | - First-trimester screening (NT screening) - Triple/Quad screening - Cell-free DNA testing/NIPT - Amniocentesis - CVS | - Triple/Quad screening - Cell-free DNA testing/NIPT | - First-trimester screening (NT screening) - Triple/Quad screening | - Trisomy 13 (Patau Syndrome) - Trisomy 18 (Edward’s Syndrome) - Trisomy 21 (Down Syndrome) | No | N/A^b^ | Free | 15 |
| MyPregnancy@NB-T  (1.0.2/March 2019) | Horizon Strategic Partners Ltd.  (hospital) | | iOS (iPhone) and Android | A hospital affiliated app that includes many articles regarding pregnancy, labor and birth, and postnatal care. | - FK grade level: 8.8 | - First-trimester screening (blood test) - First-trimester screening (NT screening) - Triple/Quad screening | - Triple/Quad screening | - First-trimester screening (blood test) - First-trimester screening (NT screening) - Triple/Quad screening | - Triple/Quad screening | - Triple/Quad screening | - Sickle cell anemia - Thalassemia - Trisomy 13 (Patau Syndrome) - Trisomy 18 (Edward’s Syndrome) - Trisomy 21 (Down Syndrome) | No | N/A^b^ | Free | 14 |
| NIPT Insights (2.1/February 2020) | Five minutes Ltd. (commercial company) | | iOS (iPhone) and Android | An educational, checklist style app that gives information about prenatal genetic testing. Savable content lets users access and discuss different options with the user’s healthcare provider. | - FK grade level: 11.4 | - First-trimester screening (NT screening) - Triple/Quad screening - Anatomy ultrasound - Cell-free DNA testing/NIPT - Amniocentesis - CVS | - First-trimester screening (NT screening) - Cell-free DNA testing/NIPT - Amniocentesis - CVS | - First-trimester screening (NT screening) - Cell-free DNA testing/NIPT - Anatomy ultrasound - Amniocentesis - CVS | - Cell-free DNA testing/NIPT | - Cell-free DNA testing/NIPT | - Trisomy 21 (Down Syndrome) - Trisomy 18 (Edward’s Syndrome) - Trisomy 13 (Patau Syndrome) | Yes (reliable source) | 5.0/5.0 | Free | 18 |
| Ovia Pregnancy Tracker: Baby Due Date Countdown (2.5.7/April 2020) | Ovia Health (commercial company) | | iOS (iPhone) and Android | A week-by-week pregnancy app with multiple graphics, articles and extensive prenatal genetic testing information. | - FK grade level: 15.1 | - Carrier screening - First-trimester screening (NT screening) - Triple/Quad screening - Cell-free DNA testing/NIPT - Amniocentesis - CVS | - First-trimester screening (NT screening) - Cell-free DNA testing/NIPT - Amniocentesis - CVS | - First-trimester screening (NT screening) - Triple/Quad screening - Cell-free DNA testing/NIPT - Amniocentesis - CVS | - Cell-free DNA testing/NIPT - Amniocentesis | - First-trimester screening (NT screening) | - Cystic fibrosis - Neural tube defect (Anencephaly) - Neural tube defect (Spina bifida) - Sickle cell anemia - Tay-Sachs Disease - Thalassemia - Trisomy 13 (Patau Syndrome) - Trisomy 18 (Edward’s Syndrome) - Trisomy 21 (Down Syndrome) | Yes (reliable source) | 4.8/5.0 | Free | 18 |
| Period Tracker by GP Apps (11.7.2/April 2020)^f^ | GP International LLC (commercial company) | | iOS (iPhone) and Android | An app includes a pregnancy mode in which users can track their pregnancy week-by-week. A pregnancy resource center tab has prenatal care and tests section that informs users about prenatal genetic testing. | - FK grade level: 9 | - First-trimester screening (blood test) - First-trimester screening (NT screening) - Triple/Quad screening - Anatomy ultrasound - Amniocentesis - CVS | - First-trimester screening (blood test) - First-trimester screening (NT screening) - Triple/Quad screening - Amniocentesis - CVS | - First-trimester screening (blood test) - First-trimester screening (NT screening) - Triple/Quad screening - Anatomy ultrasound - Amniocentesis - CVS | Not reported | Not reported | - Cystic fibrosis - Neural tube defect (Spina bifida) - Trisomy 21 (Down Syndrome) | Yes (reliable source) | 4.8/5.0 | Free ($10 annual subscription upgrade)^e^ | 17 |
| PNS Calculator  (1.02/February 2020) | California Department of Public Health (government) | | iOS (iPhone) | A calculator app that calculates the estimated date that certain prenatal genetic testing should take place. | - N/A^h^ | - First-trimester screening (blood test) - First-trimester screening (NT screening) | Not reported | - First-trimester screening (blood test) - First-trimester screening (NT screening) | Not reported | Not reported | Not reported | Yes (reliable source) | 5.0/5.0 | Free | 13 |
| Preglife: Pregnancy & Baby App (7.0.30/February 2020) | Preglife (commercial company) | | iOS (iPhone) and Android | A weekly app that features prenatal yoga and numerous articles with information on frequently asked pregnancy questions. | - FK grade level: 11.3 | - First-trimester screening (NT screening) - Triple/Quad screening - Amniocentesis | - First-trimester screening (NT screening) - Amniocentesis | - First-trimester screening (NT screening) | Not reported | Not reported | - Cystic fibrosis - Spinal muscular atrophy - Thalassemia - Trisomy 21 (Down Syndrome) | No | 4.9/5.0 | Free | 13 |
| Pregnancy + (5.8.1/January 2020) | Health & Parenting Ltd. (commercial company) | | iOS (iPhone) and Android | An app that lets users customize their app by setting appointments and writing notes for their pregnancy diary. It also provides daily information and blogs for users to read, which includes extremely limited information on prenatal genetic testing. | - FK grade level: 9.4 | - Amniocentesis - CVS | - Amniocentesis - CVS | - Amniocentesis - CVS | Not reported | Not reported | - Cystic fibrosis - Sickle cell anemia - Tay-Sachs Disease - Trisomy 21 (Down Syndrome) | No | 4.8/5.0 | Free | 12 |
| Pregnancy and Due Date Tracker^i^ (2.5.6/ March 2020) | Wachanga Inc. (commercial company) | | iOS (iPhone) and Android | A weekly app that informs users about diet, pregnancy symptoms, and fetus facts. It also includes a weight monitor, tummy growth tracker, contraction timer, and a checklist for each trimester. | - FK grade level: 10.7 | - First-trimester screening (NT screening) - Triple/Quad screening | Not reported | Not reported | Not reported | Not reported | Not reported | No | 4.8/5.0 | Free ($5.99 upgrade)^e^ | 11 |
| Pregnancy App: childbirth care and baby tracker  (1.0.0/February 2019) | Vectorr (commercial company) | | Android | A week-by-week pregnancy app with pictures, tips and minimal prenatal genetic testing information. | - FK grade level: 8.5 | - First-trimester screening (NT screening) - Cell-free DNA testing/NIPT - Amniocentesis - CVS | - First-trimester screening (NT screening) - Cell-free DNA testing - CVS | - First-trimester screening (NT screening) - Cell-free DNA testing/NIPT - Amniocentesis - CVS | Not reported | Not reported | - Trisomy 13 (Patau Syndrome) - Trisomy 18 (Edward’s Syndrome) - Trisomy 21 (Down Syndrome) | No | N/A^b^ | Free | 11 |
| Pregnancy App: what to expect week by week  (1.0.1/July 2019) | King Square Apps (commercial company) | | Android | A week-by-week pregnancy app that provides expecting mothers with tips, tools and minimal prenatal genetic testing information. | - FK grade level: 9.8 | - (Prenatal) genetic screening in general - First-trimester screening (NT screening) - Cell-free DNA testing/NIPT - Amniocentesis - CVS | - CVS | - Cell-free DNA testing - Amniocentesis - CVS | Not reported | Not reported | - Trisomy 13 (Patau Syndrome) - Trisomy 18 (Edward’s Syndrome) - Trisomy 21 (Down Syndrome) | No | 3.0/5 | Free | 11 |
| Pregnancy Calculator  (1.11/October 2016) | Androcalc (commercial company) | | Android | An app with an interactive calculator for estimating delivery date and minimal prenatal genetic testing information. | - FK grade level: 8.5 | - First-trimester screening (NT screening) - Triple/Quad screening - Anatomy ultrasound - Amniocentesis - CVS | - First-trimester screening (NT screening) | - First-trimester screening (NT screening) - Triple/Quad screening - Anatomy ultrasound - Amniocentesis - CVS | Not reported | Not reported | Not reported | No | 4.6/5.0 | Free | 10 |
| Pregnancy Calculator  (1.2/June 2018) | Mavina Seven (commercial company) | | Android | A calculator app for estimating delivery date with multiple planning resources for expecting mothers and minimal prenatal genetic testing information. | - FK grade level: 8.5 | - First-trimester screening (NT screening) - Triple/Quad screening - Amniocentesis - Anatomy ultrasound - CVS | Not reported | - First-trimester screening (NT screening) - Triple/Quad screening - Anatomy ultrasound - Amniocentesis - CVS | Not reported | Not reported | Not reported | No | 4.4/5.0 | Free | 10 |
| Pregnancy Care  (1.1.1.1.3/April 2019) | Saleha Group (commercial company) | | Android | An app with educational information about pregnancy and minimal prenatal genetic testing information. | - FK grade level: 12.4 | - First-trimester screening (NT screening) - Amniocentesis - CVS | Not reported | Not reported | Not reported | Not reported | - Cystic fibrosis - Fragile X Syndrome - Huntington’s Disease - Muscular dystrophy - Neural tube defect (general) - Trisomy 21 (Down Syndrome) | No | 4.3/5.0 | Free | 10 |
| Pregnancy Checklist  (2.2.1/March 2018) | Kigorosa UG (commercial company) | | iOS (iPhone) and Android | A checklist style pregnancy app that is grouped by trimester and has limited prenatal genetic testing information. | - FK grade level: 11.1 | - First-trimester screening (NT screening) - CVS | Not reported | - First-trimester screening (NT screening) - CVS | Not reported | Not reported | - Cystic fibrosis - Sickle cell anemia - Tay-Sachs Disease - Trisomy 21 (Down Syndrome) | No | 4.2/5.0 | Free | 12 |
| Pregnancy day by day^j^  (5.33.PD/November 2019) | HokkabazSoft (commercial company) | | Android | An app that contains comprehensive pregnancy information including day-by-day information as well as detailed and extensive prenatal genetic testing information. | - FK grade level: 8.2 | - First-trimester screening (NT screening) - Triple/Quad screening - Amniocentesis - CVS | - First-trimester screening (NT screening) - Triple/Quad screening - Amniocentesis - CVS | - First-trimester screening (NT screening) - Triple/Quad screening - Amniocentesis - CVS | - First-trimester screening (NT screening) - Triple/Quad screening - Amniocentesis - CVS | - First-trimester screening (NT screening) - Amniocentesis - CVS | - Trisomy 21 (Down Syndrome) | No | 4.7/5.0 | Free | 11 |
| Pregnancy Due Date & Fertility Calculator Tool  (2.15/September 2016) | BabymedLLC (commercial company) | | iOS (iPhone) | An app with an ovulation calculator for women trying to get pregnant as well as providing important dates for already pregnant women including information regarding some prenatal genetic tests. | - N/A^h^ | - First-trimester screening (NT screening) - Triple/Quad screening - Anatomy ultrasound - Amniocentesis | Not reported | - First-trimester screening (NT screening) - Triple/Quad screening - Anatomy ultrasound - Amniocentesis | Not reported | Not reported | Not reported | No | 4.3/5.0 | Free | 9 |
| Pregnancy Due date calculator and calendar  (1.91/March 2020) | Amicoolsoft (commercial company) | | Android | A calculator and calendar app for pregnant women with various resources. | - FK grade level: 12.7 | - First-trimester screening (NT screening) - Triple/Quad screening - Anatomy ultrasound - Amniocentesis - CVS | - First-trimester screening (NT screening) - Triple/Quad screening - Anatomy ultrasound - Amniocentesis - CVS | - First-trimester screening (NT screening) - Triple/Quad screening - Anatomy ultrasound - Amniocentesis - CVS | - First-trimester screening (NT screening) | - Triple/Quad screening | - Cystic fibrosis - Neural tube defect (Spina bifida) - Tay-Sachs Disease - Trisomy 18 (Edward’s Syndrome) - Trisomy 21 (Down Syndrome) | No | 4.6/5.0 | Free | 11 |
| Pregnancy due date tracker with contraction timer  (1.7.5/March 2020) | Pregnancy apps contraction timer, due date tracker (commercial company) | | Android | A pregnancy due date tracker app with multiple tools for expecting mothers. | - FK grade level: 10 | - First-trimester screening (blood test) - Anatomy ultrasound - Cell-free DNA testing/NIPT - Amniocentesis | - Anatomy ultrasound - Cell-free DNA testing/NIPT - Amniocentesis | - First-trimester screening (blood test) - Anatomy ultrasound - Cell-free DNA testing/NIPT - Amniocentesis | - First-trimester screening (blood test) - Anatomy ultrasound - Cell-free DNA testing/NIPT | Not reported | - Trisomy 13 (Patau Syndrome) - Trisomy 18 (Edward’s Syndrome) - Trisomy 21 (Down Syndrome) | No | 4.7/5.0 | Free | 12 |
| Pregnancy Podcast  (2.4.72/November 2019) | Pregnancy Podcast (commercial company) | | iOS (iPhone) and Android | A streaming app for recorded podcast episodes directly related to pregnancy and access to other resources with extensive prenatal genetic testing information. | - FK grade level: 10 | - Cell-free DNA testing/NIPT - Triple/Quad screening - CVS - Amniocentesis - Cordocentesis/ Percutaneous umbilical blood sampling (PUBS) | - Cell-free DNA testing/NIPT - Triple/Quad screening - CVS - Amniocentesis - Cordocentesis/ Percutaneous umbilical blood sampling (PUBS) | - Cell-free DNA testing/NIPT - Triple/Quad screening - CVS - Amniocentesis - Cordocentesis/ Percutaneous umbilical blood sampling (PUBS) | - Cell-free DNA testing/NIPT - Triple/Quad screening - CVS - Amniocentesis - Cordocentesis/ Percutaneous umbilical blood sampling (PUBS) | - Cell-free DNA testing/NIPT - Triple/Quad screening | - Trisomy 13 (Patau Syndrome) - Trisomy 18 (Edward’s Syndrome) - Trisomy 21 (Down Syndrome) | Yes (reliable source) | 4.8/5.0 | Free | 17 |
| Pregnancy Tracker- Baby Center  (3.23.3/February 2020) | BabyCenter (commercial company) | | iOS (iPhone) and Android | A week-by-week app that provides videos educating expecting mothers on pregnancy symptoms, pregnancy care and diet, and information about birth. | - FK grade level: 11.4 | - Carrier screening - Expanded carrier screening - First-trimester screening (NT screening) - Triple/Quad screening - Anatomy ultrasound - Cell-free DNA testing/NIPT - Amniocentesis - CVS | - Carrier screening - Expanded carrier screening - First-trimester screening (NT screening) - Triple/Quad screening - Cell-free DNA testing/NIPT - Amniocentesis - CVS | - Carrier screening - Expanded carrier screening - First-trimester screening (NT screening) - Triple/Quad screening - Anatomy ultrasound - Cell-free DNA testing/NIPT - Amniocentesis - CVS | - Carrier screening - Expanded carrier screening - First-trimester screening (NT screening) - Triple/Quad screening - Cell-free DNA testing/NIPT - Amniocentesis - CVS | - Carrier screening - Expanded carrier screening - First-trimester screening (NT screening) - Triple/Quad screening - Cell-free DNA testing/NIPT - Amniocentesis - CVS | - Trisomy 13 (Patau Syndrome) - Trisomy 18 (Edward’s Syndrome) - Trisomy 21 (Down Syndrome) | Yes (reliable source) | 4.8/5.0 | Free | 20 |
| Pregnancy Tracker!  (1.0.20180307-1/March 2018) | Sevenlogics, INC. (commercial company) | | iOS (iPhone) and Android | A daily pregnancy app with multiple resources for expecting mothers. | - FK grade level: 8.9 | - First-trimester screening (NT screening) | - First-trimester screening (NT screening) | - First-trimester screening (NT screening) | Not reported | Not reported | - Trisomy 21 (Down Syndrome) | No | 4.4/5 | Free | 12 |
| Pregnancy Tracker^k^  (4.11/March 2020) | What to Expect (commercial company) | | iOS (iPhone) and Android | An app with daily tips for expecting mothers including multiple articles and checklists. | - FK grade level: 12.2 | - First-trimester screening (NT screening) - Triple/Quad screening - Cell-free DNA testing/NIPT - Amniocentesis - CVS | - First-trimester screening (NT screening) - Cell-free DNA testing/NIPT - Amniocentesis - CVS | - First-trimester screening (NT screening) - Triple/Quad screening - Cell-free DNA testing/NIPT - Amniocentesis - CVS | - First-trimester screening (NT screening) - Triple/Quad screening - Cell-free DNA testing/NIPT - Amniocentesis - CVS | - First-trimester screening (NT screening) - Triple/Quad screening - CVS | - Cystic fibrosis - Neural tube defect (Spina bifida) - Sickle cell anemia - Tay-Sachs Disease - Trisomy 13 (Patau Syndrome) - Trisomy 18 (Edward’s Syndrome) - Trisomy 21 (Down Syndrome) | Yes (reliable source) | 4.8/5.0 | Free | 17 |
| Pregnancy Tracker: Baby stages, calendar & guide  (30.0.0/July 2019) | Fitness Circle (commercial company) | | Android | A pregnancy app with multiple features, including a conception calculator and pregnancy articles. | - FK grade level: 10.4 | - First-trimester screening (NT screening) - Amniocentesis - CVS | Not reported | - First-trimester screening (NT screening) | Not reported | Not reported | - Trisomy 21 (Down Syndrome) | No | 3.8/5.0 | Free | 10 |
| Pregnancy View (1.4/March 2016) | Fertility Council (commercial company) | | iOS (iPhone) | A weekly pregnancy app that gives users insight on their fetus and changes in their body during pregnancy. | - FK grade level: 9.1 | - First-trimester screening - Cell-free DNA testing/NIPT - CVS | Not reported | - First-trimester screening - Cell-free DNA testing/NIPT - CVS | Not reported | Not reported | - Trisomy 21 (Down Syndrome) | No | 2.0/5.0 | Free | 11 |
| Pregnancy Week by week^j^  (4.69.WW/September 2019) | HokkabazSoft (commercial company) | | Android | App that presents crucial information week by week for expectant mothers and provides extensive prenatal genetic testing information. | - FK grade level: 8.3 | - First-trimester screening (NT screening) - Triple/Quad screening - Cell-free DNA testing/NIPT - Amniocentesis - CVS | - First-trimester screening (NT screening) - Triple/Quad screening - Amniocentesis - CVS | - First-trimester screening (NT screening) - Triple/Quad screening - Amniocentesis - CVS | - First-trimester screening (NT screening) - Triple/Quad screening - Cell-free DNA testing/NIPT - Amniocentesis - CVS | - First-trimester screening (NT screening) - Amniocentesis - CVS | - Trisomy 21 (Down Syndrome) | No | 4.8/5.0 | Free | 12 |
| Pregnancy Week by week 2019  (1.0/October 2019) | Wafae Elkhatiri (commercial company) | | Android | A week-by-week app for pregnant women that includes information for all stages of pregnancy. | - FK grade level: 10.9 | - (Prenatal) genetic screening in general - First-trimester screening (NT screening) - Cell-free DNA testing/NIPT - Amniocentesis - CVS | - First-trimester screening (NT screening) - Cell-free DNA testing - CVS | - First-trimester screening (NT screening) - Cell-free DNA testing/NIPT - Amniocentesis - CVS | Not reported | Not reported | - Trisomy 13 (Patau Syndrome) - Trisomy 18 (Edward’s Syndrome) - Trisomy 21 (Down Syndrome) | No | 4.2/5.0 | Free | 13 |
| ProDaddy (1.0.9/January 2020) | The Prodaddy LLC (commercial company) | | iOS (iPhone) and Android | A pregnancy app designed for fathers that includes weekly tips, product recommendations, information on breastfeeding, and car seats. Weekly tips include baby names and basic doctor visits. | - FK grade level: 10.6 | - (Prenatal) genetic screening in general | Not reported | - (Prenatal) genetic screening in general | Not reported | Not reported | - Trisomy 21 (Down Syndrome) - Sickle cell anemia - Cystic fibrosis | No | 4.7/5.0 | Free | 8 |
| SBL Babies  (1.9/October 2019) | Sarah Bush Lincoln Health Center  (hospital) | | iOS (iPhone) and Android | A week-by-week app that allows users to plan out their pregnancy using checklists and trackers. It provides many articles about pregnancy, labor & delivery, and raising a baby. The pregnancy section has a pregnancy FAQ. | - FK grade level: 11.1 | - Carrier screening - First trimester screening (NT screening) - Triple/Quad screening - Cell-free DNA testing/NIPT - Amniocentesis - CVS | - Carrier screening - First-trimester screening (NT screening) - Triple/Quad screening - Cell-free DNA testing/NIPT - Amniocentesis - CVS | - Carrier screening - First trimester screening (NT screening) - Triple/Quad screening - Cell-free DNA testing/NIPT - Amniocentesis - CVS | - Cell-free DNA testing/NIPT - CVS | - Carrier screening - NT screening - Triple/Quad screening - Cell-free DNA testing/NIPT - Amniocentesis - CVS | Not reported | No | N/A^b^ | Free | 14 |
| Sprout Pregnancy (10.2/January 2020) | Med ART Studios (commercial company) | | iOS (iPhone) and Android | An app that features an interactive 3D fetus and weekly, bite-sized information regarding fetal development, and pregnancy symptoms. | - FK grade level: 10.1 | - First-trimester screening (NT screening) - Amniocentesis - CVS | - First-trimester screening (NT screening) - Amniocentesis - CVS | - First-trimester screening (NT screening) - Amniocentesis - CVS | Not reported | Not reported | - Cystic fibrosis - Neural tube defect (Spina bifida) - Sickle cell anemia - Trisomy 21 (Down Syndrome) | No | 4.7/5.0 | Free | 13 |
| Text4baby: Pregnant & New Moms  (5.1.0.17/November 2016) | Voxiva, Inc. (commercial company) | | iOS (iPhone) and Android | An app that presents pregnant women with resources around them with text-based reminders. | - FK grade level: 17.7 | - Triple/Quad screening - Anatomy ultrasound - Amniocentesis - CVS | - Amniocentesis - CVS | - Anatomy ultrasound - Amniocentesis - CVS | Not reported | Not reported | - Cystic fibrosis - Neural tube defect (general) - Trisomy 21 (Down Syndrome) | No | 3.6/5.0 | Free | 13 |
| The Bump  (5.25.0/February 2020) | The Knot Inc. (commercial company) | | iOS (iPhone) and Android | A weekly pregnancy app that provides users with many articles including prenatal genetic testing information. The app also has articles on diet and lifestyle during pregnancy. | - FK grade level: 11.4 | - Carrier screening - First-trimester screening (NT screening) - Triple/Quad screening - Anatomy ultrasound - Cell-free DNA testing/NIPT - Amniocentesis - CVS | - Carrier screening - First-trimester screening (NT screening) - Anatomy ultrasound - Cell-free DNA testing/NIPT - Amniocentesis - CVS | - Carrier screening - Triple/Quad screening - Anatomy ultrasound - Cell-free DNA testing/NIPT - Amniocentesis - CVS | - Cell-free DNA testing/NIPT - Amniocentesis - CVS | - Cell-free DNA testing/NIPT | - Cystic fibrosis - Klinefelter Syndrome - Prader-Willi Syndrome - Tay-Sachs Disease - Trisomy 16 - Trisomy 18 (Edward’s Syndrome) - Trisomy 21 (Down Syndrome) - Turner Syndrome | Yes (non-reliable sources) | 4.8/5.0 | Free | 18 |
| theAsianparent baby & Pregnancy^l^  (2.6.2/March 2020) | Tickled Media Pte Ltd (commercial company) | | iOS (iPhone) and Android | An app with multiple articles, tracking devices, support system and extensive prenatal genetic testing information for expecting and new mothers. | - FK grade level: 11.1 | - First-trimester screening (NT screening) - Triple/Quad screening - Cell-free DNA testing/NIPT - Amniocentesis - CVS - Cordocentesis/ Percutaneous umbilical blood sampling (PUBS) | - First-trimester screening (NT screening) - First-trimester screening - Triple/Quad screening - Cell-free DNA testing/NIPT - Amniocentesis - Cordocentesis/ Percutaneous umbilical blood sampling (PUBS) | - First-trimester screening (NT screening) - First-trimester screening - Triple/Quad screening - Cell-free DNA testing/NIPT - Amniocentesis - Cordocentesis/ Percutaneous umbilical blood sampling (PUBS) | - Triple/Quad screening - Cell-free DNA testing/NIPT | Not reported | - Trisomy 21 (Down Syndrome) | Yes (non-reliable source) | 4.8/5.0 | Free | 17 |
| UMC Pregnancy (1.4/December 2019) | University Medical Center (hospital) | | iOS (iPhone) and Android | The app features a 3D image of the fetus at each week of pregnancy. Each week includes health tips for the baby and pregnancy. | - FK grade level: 11.4 | - First-trimester screening (NT screening) - Amniocentesis - CVS | - First-trimester screening (NT screening) - Amniocentesis - CVS | - First-trimester screening (NT screening) - Amniocentesis - CVS | Not reported | Not reported | - Cystic fibrosis - Neural tube defect (Spina bifida) - Sickle cell anemia - Trisomy 21 (Down Syndrome) | No | 4.8/5.0 | Free | 15 |
| WebMD Pregnancy  (2.4.1/March 2020) | WebMD (commercial company) | | iOS (iPhone) | A week-by-week app that features relevant articles and tools such as a symptom tracker, contraction timer, and a Pregnancy A-Z guide. | - FK grade level: 8.9 | - Carrier screening - First-trimester screening (NT screening) - Triple/Quad screening - Cell-free DNA testing/NIPT - Amniocentesis - CVS | - Carrier screening - Triple/Quad screening - Cell-free DNA testing/NIPT - Amniocentesis - CVS | - Triple/Quad screening - Cell-free DNA testing/NIPT - Amniocentesis - CVS | - Cell-free DNA testing/NIPT - Amniocentesis - CVS | - Carrier screening - First-trimester screening (NT screening) - Triple/Quad screening | - Trisomy 13 (Patau Syndrome) - Trisomy 18 (Edward’s Syndrome) - Trisomy 21 (Down Syndrome) | Yes (reliable source) | 4.5/5.0 | Free | 18 |
| Woman’s Pregnancy (1.0.2/March 2018) | Woman’s Hospital (non-governmental organization) | | iOS (iPhone) and Android | An app has a weekly what to expect information, a to-do checklist, and FAQs for users. | - FK grade level: 14.7 | - (Prenatal) genetic screening in general - Anatomy ultrasound | Not reported | Not reported | Not reported | Not reported | - Cystic fibrosis - Sickle cell anemia - Tay-Sachs Disease - Trisomy 21 (Down Syndrome) | No | 4.8/5.0 | Free | 12 |

AQASS, App Quality Assessment Scoring System; CVS, chorionic villus screening; FK, Flesch-Kincaid; NIPT, non-invasive prenatal testing; NT screening, nuchal translucency screening; N/A, Not available

^a^The mobile apps provide the links for users to read prenatal genetic testing information from credible sources, such as the Mayo Clinic and the American Pregnancy Association.

^b^The app did not have enough customer ratings to have a customer rating score on Apple App Store or Google Play.

^c^The context of this app is the same but has a different name: “Pregnancy Parenting BabyCare - Moms Community APP” on the Google Play store.

^d^These apps were created by Providence Health & Services and had the same prenatal genetic testing information.

^e^App has a monthly subscription upgrade, but the upgrade does not affect the prenatal genetic testing information.

^f^The context of this app is the same but has a different name: “Ovulation Tracker & Fertility” on the Apple store.

^g^ “Get Pregnant: Fertility Days” did not report version or updated year and month on the Apple App Store.

^h^The app content did not have enough words to have a valid readability value.

^i^The context of this app is the same but has a different name: “Week by Week Pregnancy App. Contraction timer” on the Google Play store.

^j^These apps were created by the same developer, *HokkabazSoft*, and had the same prenatal genetic testing information.

^k^The context of this app is the same but has a different name: Pregnancy & Baby Tracker WTE on the Apple store.

^l^The context of this app is the same but has a different name: “theAsianparent Parenting Tips” on the Apple store.

^m^The theoretical range of AQASS: 0-27.
